# Thoroughness and Excellence in Dentistry

**Published:** 1873-03

**Authors:** E. S. Chisholm

**Affiliations:** Tuscumbia, Ala.


### Thoroughness and Excellence in Dentistry.

BY E. S. CHISHOLM, TUSCUMBIA, ALA.

*Extracts from an Essay Read before the Tennessee Dental Association.*

In the first place a thorough knowledge must be had of that part of the human economy to which our specialty invites us, both in its normal and abnormal conditions. Such knowledge is one of the groundworks of *excellence*. The intimate relationship existing between the teeth and the entire human system requires too a knowledge of the *whole* organism of man. Without this knowledge we can form no true estimate of the nature or extent of disease, or of the remedial agents necessary for its removal.

There are many persons who have a greater fondness for the active vocations in life, prefer a world of bustle and confusion, and could never stand the confinement incident to a pent-up office life. They could never learn to love an operation of three hours on so small an object as a carious tooth. Such persons are liable to grow discouraged with business, and dissatisfied with their profession generally, and not unfrequently combine their profession with others.

Every operation in dentistry, as in everything else, should be thorough. We should never begin any work without a determination to complete it. Each separate part of an operation must be perfect within itself, the whole becoming a complete body. Work well done is twice done. We are too much disposed to slight work because at first sight it seems not to be absolutely important. Neglect of small matters often leads to the greatest troubles. Who has not lost a filling by failing to trim away, at some remote point, the extruding ends of gold, thereby leaving a lever for its dislodgment, or "built upon a sandy foundation" by taking hurriedly an impression? Even though trouble should fail to come of such indifferent work, such a course leads to habitual neglect, and we degenerate insensibly into bad operators.

In operative dentistry there is no point which should receive a closer consideration than excavation; and though I may not mention anything new, yet if I but reiterate a good thought I will be repaid. This process shouid be thorough, as otherwise no excellent operation can be performed. Admitting the susceptibility of teeth, to morbid impressions in peculiar constitutional habits, yet in a very great number of cases, teeth can be saved even though subject to such influences. My general mode of excavation is about as recommended in the following:

1st. *Trim the enamel well to the depth of all disintegrated dentine, and remove thoroughly.* Sometimes the enamel on the grinding surfaces of the bicuspids and molars is very hard, and even after they appear well excavated, there is a line of decay running between the enamel and dentine, and not unfrequently pockets of softened tooth under the edge of the enamel. Chisels are the best instruments for this purpose; but should the cavity be uniform in shape a burr is the best.

2d. *Never excavate, leaving fissures untouched.* These are more frequently found on the grinding ends of the tooth, but often extend to the buccal or lingual surfaces. Though small, they serve as inroads to undermine your filling. They should be thoroughly rounded with a small drill at their extremities, and their walls cut perpendiculariy, and sufficiently large to admit of good filling. To fill these indifferently would be as improper as to fill the main cavity imperfectly.

3d. Do as little undercutting and dovetailing as will insure good attachments to the plug. It exposes the parietes to fracture, and often renders the nice adaptation of the gold to the walls exceedingly difficult.

4th. *Trim especially well the cervical portion of the cavity.* The proximity of the teeth renders judicious excavation at such places exceedingly difficult; and often when the cavity is even with or below the gum, a constant discharge of blood exudes from the gum very much to our annoyance. The want of a firm basis upon which to begin the filling would preclude the possibility of successful work, and if again attacked with caries, would be the first point of exposure of the nerve, the patient having no knowledge of the decay till aching begins.

5th. *Trim the wall of the cavity as smooth as possible.* Close and thoroughly adjust is the grand secret of success, and this cannot be done but by having the walls sufficiently smooth to admit of its close adaptation.

6th. In all cavities upon the sides of the teeth, where there is the least danger of the walls breaking in by mastication, cut down through the grinding surface, and fill all together. If this precaution is not observed, the tooth will by degrees be fractured, and leave the plug to become dislodged, and ultimately drop out.

7th. Bevel the edge of every cavity at an angle of about forty-five degrees. This will frequently prevent shivering the edges in the finishing up of a malleted filling, or from fracture from mastication when on the grinding surface of the teeth.

8th. Thoroughly cut away all decay in all cavities, unless liable to expose the nerve. When decays are deep there may be only a thin lamina of bone or dentine over the nerve, and to cut through would bring about a much more formidable difficulty. To thus leave the decay, and touch the surfaec with creosote, will frequently do much towards hardening the structure. Teeth have been examined years after such treat-treatment, and found much improved in solidity.

With these suggestions, I leave the suject of excavation with you, by remarking that perfect contact of the filling material with the walls of a cavity in all its parts, if the above prerequisites are observed, will in nearly every instance be a preventive of further decay, it matters not what the constitutional influences are.

